# More robust evidence for the efficacy of lithium in the long-term treatment of bipolar disorder: should lithium (again) be recommended as the single preferred first-line treatment?

**DOI:** 10.1186/s40345-014-0017-6

**Published:** 2015-01-31

**Authors:** Willem A Nolen

**Affiliations:** Department of Psychiatry, University Medical Center Groningen, University of Groningen, Hanzeplein 1, 9713 GZ Groningen, The Netherlands

**Keywords:** Lithium, Bipolar disorder, Long-term treatment, Guideline

## Abstract

With two recent systematic reviews and meta-analyses on the efficacy of lithium compared to placebo and other treatment options, it can now be concluded that lithium is the only drug that has been shown efficacious in the prevention of any mood episodes, manic episodes and depressive episodes in randomised trials not enriched for prior response to and tolerance of lithium. It is argued that lithium should be recommended as the single preferred first-line drug in the long-term treatment of bipolar disorder.

## Correspondence

Among the many options for the long-term treatment of bipolar disorder, lithium is the oldest drug. After its initial use by the Danish neurologist Lange in the nineteenth century and its subsequent discovery by the Australian psychiatrist Cade in 1949, the efficacy of lithium was finally established by Schou and his Danish colleagues in the 1960s which led to its registration in Europe and the US as well as many other countries in the 1970s (Bech 2006). Since then, several other drugs have become available as alternatives, of which the anticonvulsants carbamazepine, valproate and lamotrigine and several atypical antipsychotics have also been registered for the long-term treatment of bipolar disorder. Subsequently, all registered drugs also found their place in guidelines, supported by data from randomised placebo-controlled clinical trials and systematic reviews and meta-analyses, including a meta-analysis on lithium versus placebo which found that lithium did prevent any mood episodes and manic episodes, but was not significantly better than placebo in the prevention of depressive episodes (Geddes et al. 2004).

Overall, these data led to recommendations in most guidelines for more than one drug as first-line treatment for the long-term treatment of bipolar disorder. For instance, the latest US APA guideline from 2002 (!) recommends lithium or valproate and as possible alternatives lamotrigine, carbamazepine, oxcarbazepine and ongoing antipsychotic treatment (American Psychiatric Association 2002); the UK NICE guideline from 2006 lithium, olanzapine or valproate (National Institute for Health and Care Excellence (NICE) 2006); the updated WFSBD guideline from 2012 ‘*based on strongest level of evidence for efficacy and a good risk*-*benefit ratio*’ aripiprazole, lamotrigine, lithium and quetiapine (Grunze et al. 2013); and finally the updated Canadian CANMAT and ISBD guideline from 2013 lithium, valproate, olanzapine, quetiapine, lamotrigine, risperidone long-acting injection (LAI) and ziprasidone (Yatham et al. 2013).

In 2011, the so far largest long-term study on the efficacy of lithium was published (Weisler et al. 2011). In this double-blind trial, bipolar I patients who had responded to and had tolerated quetiapine were randomised to continue with quetiapine or to switch to lithium or placebo. The results showed that both quetiapine and lithium did prevent any mood episodes, manic episodes and depressive episodes. Quetiapine was also more effective than lithium (in the prevention of any mood episodes and depressive episodes), but this result should be interpreted with caution as the study design was enriched for quetiapine (see above) but not for lithium. Only the WFSBP and the CANMAT/ISBD guidelines did include the Weisler et al. 2011 trial, while the WFSBP guideline also concluded that lithium was the only drug with the strongest level of evidence (level A for any mood episodes, manic episodes and depressive episodes) in non-enriched samples.

Two recent meta-analyses further looked at the evidence of lithium. In this journal, Severus et al. (2014) published an update and extension of the meta-analysis by Geddes et al. from 2004. With seven placebo-controlled trials, i.e. two more than in 2004 including the Weisler et al. 2014 trial, they now found that lithium not only prevents any mood episodes and manic episodes but also prevents depressive episodes. This finding was further supported by a network meta-analysis including 33 randomised controlled trials examining 17 treatments (both monotherapies and combination therapies) by Miura et al. (2014). Of the monotherapies, only lithium and quetiapine were found to prevent any mood episodes, manic episodes and depressive episodes when compared to placebo, with the notion that the evidence for lithium is based almost completely on trials with a non-enriched design and for quetiapine only on trials with enriched designs. Both meta-analyses also looked at tolerance of lithium (more dropouts due to adverse events with lithium than with placebo) and at overall satisfaction with treatment (more completers with lithium than with placebo).

The conclusions of both meta-analyses are rather similar. Severus et al. state that ‘*with no other drug available having such ample and consistent evidence for its efficacy, lithium remains the most valuable treatment option in this indication*’, and Miura et al. conclude that ‘*lithium seems to be the most reasonable candidate for a first*-*line option for the long*-*term treatment of bipolar disorder*’. This raises the question whether lithium should indeed be recommended as the single preferred first-line treatment. Indeed, the recent 2014 update of the NICE guideline (with their own meta-analyses) recommends to ‘*offer lithium as a first*-*line, long*-*term pharmacological treatment for bipolar disorder*’ , while regarding the other alternatives, the guideline continues as follows: ‘*if lithium is ineffective, consider adding valproate or if lithium is poorly tolerated, or is not suitable (for example, because the person does not agree to routine blood monitoring), consider valproate or olanzapine instead or, if it has been effective during an episode of mania or bipolar depression, quetiapine*’. Thus, despite these considerations, the National Institute for Health and Care Excellence (NICE) (2014) guideline recommends lithium as the first option.

One of the major limitations of all meta-analyses is that while they looked at efficacy, tolerability and overall satisfaction, their scope was not more than 2 years. However, when choosing a drug for long-term if not life-long treatment, one should look at efficacy and safety not only during the first years but also thereafter. The use of lithium over more than 10 years is associated with the risk of kidney dysfunction and the use of atypical antipsychotics with metabolic syndrome and an increased mortality risk due to cardiovascular problems. Although it has not yet been established how these late adverse effects have an impact on the long-term safety of lithium and the atypical antipsychotics as its major alternatives, the assumption is that also over 10 or more years the safety of lithium is at least in balance with that of most atypical antipsychotics. However, it would be interesting to see studies in patients with bipolar disorder looking at mortality during treatment with lithium and its alternatives over more than 10 years such as has already been done with clozapine versus other antipsychotics in patients with schizophrenia (Tiihonen et al. 2009).

In the meantime, I concur with the conclusions of both meta-analyses supporting the recommendation in the NICE guideline that lithium, given its evidence base from trials with a non-enriched design and its relative safety, should be the single first-line treatment. Whether or not this conclusion will be supported by most experts in the field of lithium will become clear in 2016 when the recently started ISBD task force on the use of lithium will present its report.

## Abbreviations

APA: American Psychiatric Association
CANMAT: Canadian Network for Mood and Anxiety Treatments
ISBD: International Society for Bipolar Disorders
NICE: National Institute for Health and Care Excellence
WFSBP: World Federation of Societies of Biological Psychiatry
